# Real Implication of Fertility-Sparing Surgery for Ovarian Cancer: Reproductive Outcomes

**DOI:** 10.3390/diagnostics14131424

**Published:** 2024-07-03

**Authors:** Marta Heras, Maria Alonso-Espias, Octavio Arencibia, Lucas Minig, Lola Marti, Maria Dolores Diestro, Juan Cespedes, Isabel Niguez, Blanca Gil-Ibañez, Berta Diaz-Feijoo, Antoni Llueca, Claudia Rosado, Sara Iacoponi, Carlos Lopez de la Manzanara, Sara Morales, Maria Jose Fernandez-Galguera, Ana Cano, Mikel Gorostidi, Ignacio Zapardiel

**Affiliations:** 1 Gynecology Department, Hospital Universitario Santa Cristina, 28009 Madrid, Spain; marta.heras@salud.madrid.org; 2 Gynecologic Oncology Unit, La Paz University Hospital, 28046 Madrid, Spain; 3 Gynecology Department, Hospital Universitario Materno Infantil, 06010 Las Palmas de Gran Canaria, Spain; 4 Gynecology Department, IMED Hospitales, 46100 Valencia, Spain; 5 Gynecology Department, Hospital Universitario Bellvitge, 08907 Barcelona, Spain; 6 Gynecology Department, Hospital Universitario de Donostia, 20014 San Sebastian, Spainmgorostidi@sego.es (M.G.); 7 Gynecology Department, Hospital Virgen de la Arrixaca, 30120 Murcia, Spain; 8 Gynecology Department, Hospital Universitario 12 de Octubre, 28041 Madrid, Spain; 9 Institute Clinic of Gynecology, Obstetrics and Neotatology, Hospital Clinic de Barcelona, 08007 Barcelona, Spain; 10 Gynecology Department, Hospital de Castellón, 12004 Castellón, Spain; 11 Gynecology Department, Hospital de Mataró, 08304 Barcelona, Spain; 12 Gynecology Department, Hospital Quirón Madrid, 28223 Madrid, Spain; 13 Gynecology Department, Hospital General Universitario de Ciudad Real, 13005 Ciudad Real, Spain; 14 Gynecology Department, Hospital Universitario Infanta Leonor, 28031 Madrid, Spain; 15 Gynecology Department, Complejo Asistencial de Zamora, 49022 Zamora, Spain; 16 Gynecology Department, Hospital Universitario Fundación Alcorcón, 28922 Madrid, Spain

**Keywords:** ovarian cancer, fertility, pregnancy

## Abstract

Background: to prove the effectivity of fertility-sparing procedures in early-stage ovarian cancer by assessing pregnancy rates and obstetrical outcomes. Methods: we performed a retrospective multicenter study among 55 Spanish hospitals, collecting patients from 18 to 40 years old with diagnosis of early-stage ovarian cancer, epithelial (EOC) or non-epithelial (non-EOC), from January 2010 to December 2019. Data on the use of assisted reproductive techniques, pregnancy attempts and obstetrical outcomes were collected. Results: a total of 150 patients met inclusion criteria, 70 (46.6%) EOC and 80 (53.4%) non-EOC. Pregnancy attempts were reported in 51 (34%) patients, with 42 (28%) patients carrying the pregnancy to term. Among them, 30 (71.4%) underwent surgery alone and 12 (28.6%) had additional postoperative chemotherapy. A total of 32 (76.1% patients) had spontaneous pregnancies and 10 (23.9%) required in vitro fertilization. There was only one (2.4%) complication reported. Vaginal delivery was reported in twenty-nine (69%) patients and cesarean section in five (11.9%) patients. Conclusions: fertility-sparing management for ovarian cancer seems to be an option with proven good pregnancy rates and low complications. The selection of patients must consider strict criteria in order to maintain a good prognosis.

## 1. Introduction

As with many other tumors, ovarian cancer is more frequently diagnosed in post-menopausal women, although up to 10% of cases are reported in patients under 50 years old [1]. Depending on histology, it is divided into two main subgroups which are epithelial ovarian cancer (EOC) and non-epithelial ovarian cancer (non-EOC); the frequency of each subtype is influenced by age at diagnosis [2]. EOC cases represent around 90% of the total amount of ovarian malignant tumors and are more frequent in older patients, while younger patients have more frequently non-EOC and early-stage tumors [3,4]. Non-EOC cases are usually less aggressive tumors that confer excellent overall survival [5,6]. When an ovarian mass is evidenced, the degree of suspicion is important to establish appropriate management; however, histological analysis is mandatory to confirm the diagnosis of cancer.

Treatment for ovarian cancer is usually multidisciplinary, including surgery and chemotherapy, and it is the cause of premature menopause and loss of fertility in young patients. In general, young patients with ovarian cancer have higher overall survival rates than old patients, since their tumors tend to be less aggressive, are more often presented in the early stage and the surgical treatment is not limited by the patient’s morbidities [7]. Moreover, social changes have led to a delay on childbearing, so it is important to consider that many young patients might not have fulfilled their maternity wishes prior to cancer diagnosis and will therefore demand fertility preservation whenever possible [8]. These changes have encouraged oncology specialists to develop conservative procedures for ovarian cancer treatment, which may allow a pregnancy [9]. Conservative treatments are also based on surgery and chemotherapy, and they have to offer similar relapse rates and overall survival than standard procedures; their safety is proven when the selection of patients is adequate [10]. Fertility-sparing surgery (FSS) has become the standard procedure for young patients with non-EOC, since the tumors are usually unilateral and, even in advanced stages, their response to chemotherapy is high, so they can be amenable to neoadjuvant chemotherapy followed by FSS [11]. On the contrary, management of EOC is challenging, since the tumors are aggressive with high rates of recurrence, so the use of conservative procedures must be after adequate selection of patients.

The role of surgery on fertility preservation is clear, since it is compulsory to maintain the uterine body and, in most cases, at least part of the contralateral ovary to keep the possibility for pregnancy. The role of chemotherapy alone is less clear, since most studies that show the influence of chemotherapy combined with radiotherapy are heterogeneous studies, including male and female patients, a wide variety of tumors and a wide age range, from childhood to fertile age [12,13]. Data on only gynecological cancer patients, such as ovarian cancer, are very limited; there is also scarce information about the factors that could affect the pregnancy achievement. Ovarian damage due to chemotherapy seems to be agent, dose and age dependent [14,15]. Considering agents, alkylating agents have the most detrimental effects on the ovary; platinum agents are considered alkylating-like agents and present moderate induced ovarian damage [16,17]. Cisplatin reduces follicle counts, estradiol and antimüllerian hormone (AMH) productions [18]. Paclitaxel damages mature oocytes, with transitory reduction of reproductive potential [19]. Doses have shown a deleterious effect on fertility, showing that the more cycles of chemotherapy, the more delay on pregnancy and need for reproductive assisted techniques [20]. Non-alkylating and non-gonadotoxic agents only affect fertility if given in very high doses [21]. Age and previous ovarian reserve also play a role in the degree of gonadotoxicity due to chemotherapy, and patients younger than 30 years old recover normal AMH concentrations faster and more frequently than older patients, and also have increased oocyte quality [22,23,24].

Nonetheless, the performance of conservative procedures for ovarian cancer does not guarantee pregnancy; furthermore, cancer survivors that underwent FSS have shown lower pregnancy rates than controls [25,26]. After completion of treatment, attempt of pregnancy must not be immediate since ovaries are the most sensitive organ to chemotherapy in the female reproductive system, and deleterious effects on cells may last [27].

Our aim was to analyze the implications and real morbidities of fertility-sparing treatment for ovarian cancer, including rates of pregnancy, proportion of spontaneous pregnancies and obstetrical outcomes, in order to identify risk factors for poor pregnancy outcomes.

## 2. Materials and Methods

We carried out a multicenter retrospective national study including young patients that underwent conservative treatment after diagnosis of ovarian cancer from January 2010 until December 2019. Fifty-five institutions collected patients after approval of their local ethical committee. The inclusion criteria included the following: patient’s age from 18 to 40 years old, histological diagnosis of malignancy, any histological type, early-stage of the disease (FIGO stages I or II) and performance of fertility-sparing procedures. Exclusion criteria were the following: non-invasive tumors, patients outside the age range, stages III or IV, or performance of standard staging surgery. All patients that met inclusion criteria for conservative management and wished to undergo fertility-sparing treatment were informed of the potential risks of this management, besides the possibility of choosing radical surgery as standard of care, with the subsequent loss of fertility. All patients signed an informed consent before surgery.

The surgical route included laparotomy, laparoscopy, or robotics. The selection of the surgical approach was based on patient characteristics and surgical team experience. Extension of surgical procedures was different depending on tumor histology. FSS for EOC was a standard procedure and consisted of unilateral adnexectomy, bilateral pelvic lymphadenectomy, para-aortic lymphadenectomy, omentectomy, peritoneal cytology and peritoneal biopsies. Non-EOC surgical treatment was more heterogeneous between institutions, but avoidance of pelvic and para-aortic lymphadenectomy was permitted if the absence of enlarged nodes was assessed; it was also compulsory to assure access to postoperative chemotherapy if needed. The minimal treatment considered in these cases was unilateral salpingo-oophorectomy or cystectomy with inspection of the abdominopelvic cavity, depending on the decision of each hospital´s tumor board. The uterine body was preserved in all cases. Chemotherapy was administered postoperatively depending on the local protocols and based on published guidelines. The standard chemotherapy regimens used included cisplatin and paclitaxel for EOC, and bleomycin, cisplatin and etoposide for non-EOC. All patients were free of disease at the time of the pregnancy attempt.

Pregnancies were obtained either spontaneously or after assisted reproductive techniques (ARTs). The need for ARTs was considered according to the individual center protocol including patient´s age, ovarian reserve and immediacy of maternity. Gestational control was carried out on high-risk obstetric units with concomitant follow-up for ovarian cancer. The route of delivery was based on obstetrical characteristics only, without being influenced by personal history of cancer nor fear of birth trauma.

**Statistical analysis.** Kolmogorov–Smirnov and Saphiro–Wilk tests were used to evaluate the normal data distribution of the collected variables. Absolute frequencies and proportions were used as summary statistics for categorical variables, while the mean (standard deviation) or median and interquartile (IQ) range were used for continuous variables. Bivariate analysis was performed using Fisher´s exact test for categorical variables and the Student´s test for continuous variables between groups. The time to pregnancy was plotted using the Nelson Aalen cumulative hazard estimates. The log-rank test was used to assess the time-to-event differences between groups by univariate analysis. All the tests were two-sided and alpha error was set at 5%. The analyses were performed using the software STATA 15.1 (StataCorp LLC, College Station, TX, USA).

## 3. Results

A total of 150 patients were included in the study, including 70 (46.6%) with EOC and 80 (53.4%) with non-EOC. The mean age of the patients was 28.57 years old, with a mean tumoral size of 108.2 cm. Considering FIGO stage, 143 (95.3%) patients were stage I, divided into 99 (66%) stage IA, 5 (3.3%) IB, 30 (20%) IC1, 6 (4%) IC2 and 3 (2%) IC3. The remaining 7 (5%) patients were stage II, with 2 (1.3%) patients being stage IIA and 5 (3.3%) IIB. All patients underwent conservative procedures, which included surgery in all cases, followed by adjuvant chemotherapy if recommended based on clinical guidelines. Fertility-sparing surgery was not a standard procedure, since the extension of surgery was highly influenced by histology; FIGO stage and institution´s protocol also played a role in its planification. After surgery, 51 (34%) patients required adjuvant chemotherapy, corresponding to 19 (37.2%) patients in the EOC group and 32 (62.8%) patients in the non-EOC group. None underwent neoadjuvant chemotherapy.

After diagnosis, patients were explained the possibility of undergoing standard treatment or conservative procedures, as well as the mandatory loss of possibility of childbearing if standard procedures were carried out. The majority of patients that underwent fertility-sparing treatment did not have children before their cancer diagnosis; only forty-one (27.3%) patients had, of whom 24 (58.5%) patients had just one child and the remaining 17 (41.5%) had more than one. All patients were also explained the potential diminution of fertility due to treatment, but only 20 (13.3%) underwent oocyte preservation before cancer treatment.

The mean follow-up time was 60.19 months, considered as months between the end of treatment and the last medical appointment.

**Figure 1:**
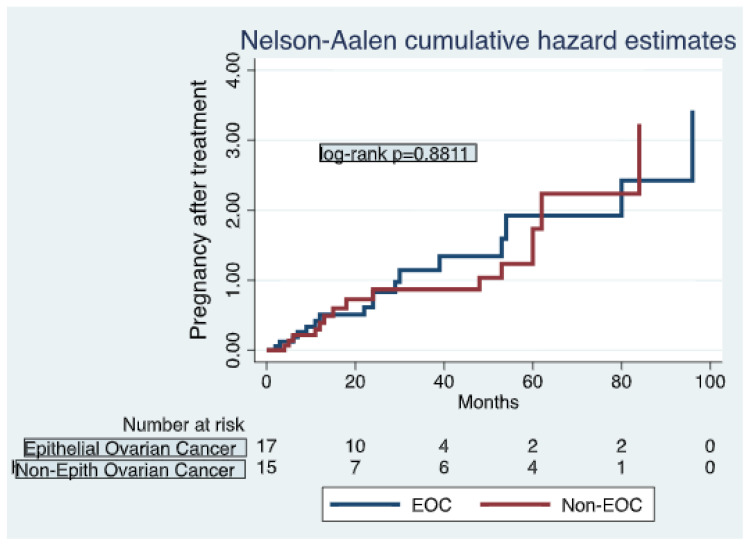
Pregnancies after completion of treatment, based on histology.

Finally, 51 (34%) patients of the 150 tried to conceive after fertility-sparing management. Among patients who tried gestation, 42 (82.4%) patients obtained 51 term pregnancies. This included thirty-three patients who had one pregnancy and nine patients who had two. The remaining nine (17.6%) patients had unsuccessful gestation attempts, of whom four (7.8%) had miscarriages and five (9.8%) reported cases of infertility. Only one (2%) patient had offspring prior to their cancer diagnosis and completed maternity wishes after treatment. Pregnancy rates seemed to be higher in the EOC group compared to the non-EOC patients, with 25 pregnancies in the EOC group and 17 in the non-EOC group (16.6% vs. 11.3% of the total amount of patients that preserved fertility), although no significant differences were observed (Figure 1). The pregnancy rate was higher in patients who underwent minimally invasive surgery compared to those who underwent laparotomy. We observed 23 pregnancies reported in patients treated with MIS vs. 19 pregnancies in patients with laparotomy (54.8% vs. 45.2%).

Considering the treatment performed among the patients who obtained term pregnancies, 31 (73.8%) patients underwent only surgery and 11 (26.2%) underwent surgery and adjuvant chemotherapy. Adjuvant chemotherapy was more frequent in non-EOC patients than in EOC patients, with eight patients in the non-EOC group undergoing adjuvant chemotherapy vs. three patients with EOC.

Pregnancy rates were also influenced by FIGO stage: among the 42 patients that obtained pregnancy, thirty (71.4%) were stage IA, one (2.4%) IB, eight (19%) IC1, one (2.4%) IC2, one (2.4%) IC3 and one (2.4%) IIB; some patients had more than one child. Comparison among EOC and non-EOC is shown in Table 1.

The only complication reported during pregnancy was gestational diabetes and the pregnancy reached the term. Vaginal delivery was the most frequent route of delivery and the percentage of cesarean section was not higher than in routine obstetric practice. Concerning recurrences, twelve (8%) patients presented a recurrence after FSS for ovarian cancer; only one (0.66%) presented a relapse after pregnancy, which was a non-EOC FIGO stage IC3 case. The patient had an abdominal recurrence 76 months after cancer diagnosis, which was treated with surgery and chemotherapy with complete response, and she was free of disease at the point of data collection, 123 months after initial diagnosis.

## 4. Discussion

**Figure 2:**
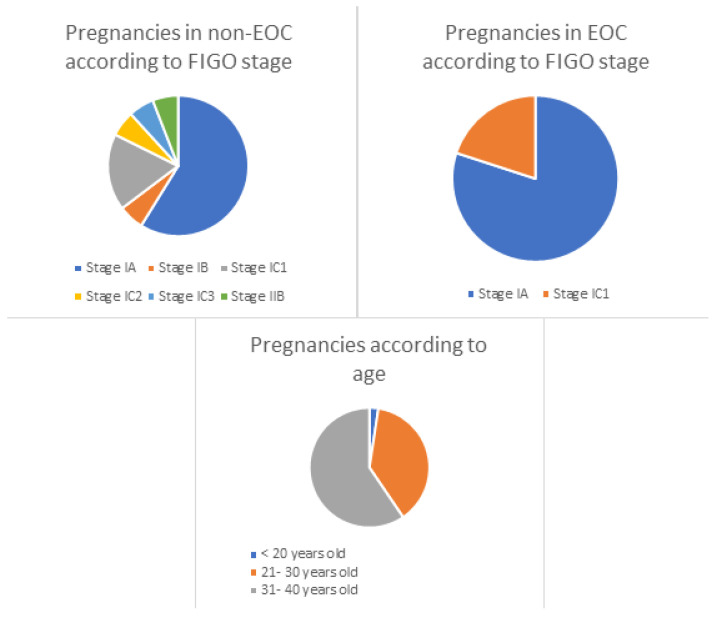
Pregnancy rates based on patients’ histology, age and FIGO stage.

Fertility-sparing management has been developed as a novel option for ovarian cancer treatment in young patients, allowing the accomplishment of maternity wishes in patients that, otherwise, would have undergone radical surgery with the subsequent loss of that possibility. Conservative procedures, such as unilateral salphingo-oophorectomy in early stages, have shown good pregnancy rates and low pregnancy complications. In our study, 42 patients achieved at least one term pregnancy, representing 28% of successful pregnancies among all patients who underwent FSS. However, attempt of pregnancy was reported in only 51 of our patients, so the actual fertility rate was 82.3%. Pregnancy rates were influenced by multiple factors such as histology, FIGO stage and age at diagnosis (Figure 2). Patients with EOC had higher pregnancy rates than patients with non- EOC. Considering FIGO stage, patients with stages IA, IB and IC1 also had more pregnancies than patients with stages IC2 and IC3. In addition, gestations were more frequent in patients from 31 to 40 years old, followed by those from 21 to 30 years old.

There are many considerations that may explain the influence of these factors on the number of pregnancies reported. Histology has a great impact on fertility rates, but it is probably highly influenced by patients’ age and the special features of non-EOC regarding treatment. In our series, of the 42 term pregnancies reported, 25 were in patients with EOC and 17 were in patients with non-EOC (59.5% vs. 40.5%, respectively). Patients diagnosed with EOC were elder than patients with non-EOC, and their maternity wishes were probably more immediate. Also, patients in the EOC group were more often offered a completion of treatment with hysterectomy, so they had to advance their search for pregnancy in order to undergo complete surgery as soon as possible. FIGO stage also plays a role in achievement of gestation. Stage I tumors can be subdivided into low-risk and high-risk, considering low-risk patients those with stage IA, IB or IC1 and high-risk patients those with stage IC2 and IC3. We report thirty-nine (92.9%) patients with pregnancies in stages IA, IB and IC1; on the other hand, only three (7.1%) patients got pregnant with stages IC2, IC3 and II, showing the influence of prognosis according to risk of recurrence in the search for motherhood. In addition, since even in young ages an advanced stage is very common, and fertility preservation is not indicated for stage III disease, this option of fertility preservation in ovarian cancer has a limited role for selected patients. The number and attempt of pregnancies in the low-risk patients’ group was much higher than in the high-risk patients group. When EOC is low risk and confined to the ovary, FSS can be offered with similar recurrence rates (RRs) and overall survival (OS) than standard staging surgery (SSS) [28]. Published data also support feasibility of FSS in early-stage high-risk cases, since; although recurrences and deaths are more frequent than in low-risk cases, prognosis is similar comparing SSS and FSS [29]. Considering FIGO stage and its subdivision among risks for recurrence, there were more pregnancies reported on low-risk patients. This is probably influenced by the patient’s self-awareness of their prognosis and risk for recurrence, so patients in the high- risk group are less willing to assume a recurrence if they have offspring in their care. They might also be more afraid of an increased risk of relapse due to the influence of a pregnancy, and would therefore avoid it. Finally, age at ovarian cancer diagnosis also influenced pregnancy rates. Patients from 31 to 40 years old had the highest rate of gestations with 25 (59.5%) patients with reported pregnancies. Sixteen (38%) patients aged from 21 to 30 years old had pregnancies and one (2.5%) patient was under 20 years old. The current propensity of having children at later ages seems multifactorial, influenced by social and economic factors but also by psychological reasons. Elder patients are more aware of the passage of time and the biological decrease in their fertility, and might also have had maternity wishes prior to cancer diagnosis, so, when allowed, they tend to accelerate the search for pregnancy.

It is remarkable that, although the objective of treatment was the preservation of the possibility of having children, cancer survivors have fewer pregnancy attempts reported than patients of the same age group that have not been diagnosed with cancer [30,31]. The diminished rate of pregnancy of cancer survivors does not seem to be only caused by diminished fertility, but of multifactorial causes, and it is difficult to know if it is due to radiotherapy, chemotherapy, both or none of them [32,33]. Psychological reasons, such as concerns about increasing complications during pregnancy, hereditary diseases for the offspring or worse self-cancer prognosis among patients achieving pregnancy also play a role in reduced fertility. Studies showed that only 60% of patients have reported pregnancy attempts after conservative treatment, or even lower rates; in our study only 51 of 150 patients had reported pregnancy attempts (34%) [34,35]. Those rates are more diminished in patients with high-risk tumors compared to low-risk tumors and patients who had children before their cancer diagnosis [36].

**Figure 3:**
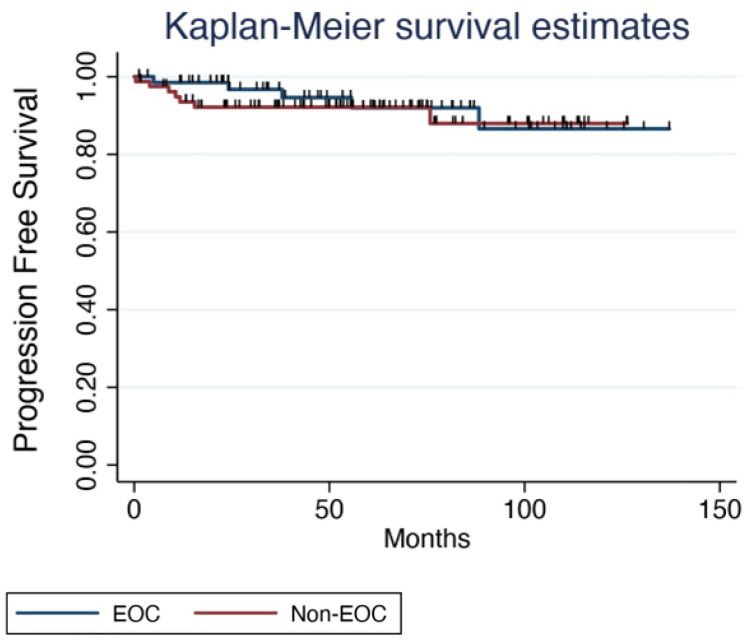
KM survival estimates according to histology.

Pregnancy outcomes have not been influenced either by type of conservative surgery or need for adjuvant chemotherapy. Miscarriages were reported in four (7.8%) cases, which is lower than usual. Route of delivery was determined by obstetrical factors only, with no higher percentage of cesarean section than in routine obstetric practice. As for pregnancy complications, only one case of gestational diabetes was described, which is a frequent complication in standard pregnancies, without higher rates described in cancer survivors in our study. Published data considering implications of cancer treatments in pregnancy complications are heterogeneous. Cancer survivors have increased rates of endocrine, cardiac or pulmonary disorders, due to treatment but also to increased age; those disorders are probably related to increased morbidities in pregnancy [37]. Women who attempt pregnancy after cancer have higher risk of gestational morbidities such as abortion, preeclampsia, gestational diabetes and cesarean section compared with the non-cancer group, but those factors are also strongly related to age [38,39]. Neonatal morbidities are also increased, such as intrauterine growth restriction, low weight at birth, preterm delivery and low Apgar score [40]. Cesarean delivery is more frequent in patients diagnosed with cancer, although the explanation needs further investigation; it may be due to the increase in pregnancy morbidities in those patients or to fear of birth trauma. Furthermore, pregnancies do not impoverish patients’ self-prognosis nor have influence on recurrences. We report 12 (8%) recurrences after fertility-sparing procedures, and only one (0.66%) was after a term pregnancy and delivery. The patient had a complete response to treatment. Finally, death due to ovarian cancer was reported in four patients, two with EOC and two with non-EOC (Figure 3); none of them had pregnancies.

There are a number of limitations in our study that need to be explained. First, the heterogeneous collection of data meant that attempt of pregnancy was not registered in every patient, so there are probably more patients with unsuccessful pregnancy wishes than reported. Second, being a multicenter study, the extension of surgery and surgical route may be different between hospitals, although it was always based on guidelines and approved by a multidisciplinary committee. To conclude, the large number of factors that impact the search for motherhood make it very difficult to establish which patients really had infertility from those who, besides undergoing fertility-sparing treatment, finally decide not to have children.

## 5. Conclusions

Fertility-sparing management for ovarian cancer seems to be a safe option with proven good pregnancy rates and low complication profile. Pregnancy rates among patients that have reported active pregnancy search are high, although many cancer survivors do not try to get pregnant. Accomplishment of pregnancy or use of assisted reproductive techniques does not seem to decrease the oncological safety of conservative procedures. As for pregnancies, the rate of complications was not higher than usual in cancer survivors.

## Figures and Tables

**Table 1:** Comparison among epithelial and non-epithelial ovarian cancer.

	Epithelial Ovarian Cancer	Non-Epithelial Ovarian Cancer
**Number of patients**	70	80
**Age**	Mean 30.3	Mean 27.2
**Pregnancies prior to cancer diagnosis**	Mean: 0.26	Mean: 0.54
**Number of pregnancies after cancer**	25 (35.7%)	17 (21.25%)
**Surgical approach** **Laparotomy** **Minimally invasive**	36 (51.4%) 34 (48.6%)	43 (53.7%) 37 (46.3%)
**Type of pregnancy** **Natural** **Assisted reproductive techniques** **Missing data**	17 (68%) 7 (28%) 1 (4%)	14 (82.4%) 3 (17.6%) 0
**Pregnancy complications**	0 (0.0%)	1 (1.25%)
**Newborn weight**	Mean: 3171 g	Mean: 3267 g
**Type of delivery** **Vaginal** **Cesarean section** **Missing data**	17 (68%) 4 (16%) 4 (16%)	12 (70.5%) 1 (5.8%) 4 (23.7%)
**Recurrences**	5 (7.14%)	7 (8.75%)
**Completion of histerectomy after pregnancy attempt**	9 (12.8%)	4 (5%)

## Data Availability

The data presented in our study were obtained due to an online shared form that was downloaded into an excel database. Content data are encrypted.

## References

[1] Kajiyama H., Suzuki S., Yoshikawa N., Kawai M., Mizuno K., Yamamuro O., Nagasaka T., Shibata K., Kikkawa F. (2019). Fertility-sparing surgery and oncologic outcome among patients with early-stage ovarian cancer ~propensity score- matched analysis~. BMC Cancer.

[2] Ali A.T., Al-Ani O., Al-Ani F. (2023). Epidemiology and risk factors for ovarian cancer. Menopausal Rev..

[3] Jayson G.C., Kohn E.C., Kitchener H.C., Ledermann J.A. (2014). Ovarian cancer. Lancet.

[4] Cheung A., Shah S., Parker J., Soor P., Limbu A., Sheriff M., Boussios S. (2022). Non-Epithelial Ovarian Cancers: How Much Do We Really Know?. Int. J. Environ. Res. Public Health.

[5] Zhang C., Xi X. (2023). Clinicopathological Features and Survival Trends of Non-Epithelial Ovarian Cancer: Analysis of the Surveillance, Epidemiology, and End Results (SEER) Database. Oncol. Res. Treat..

[6] Brown J., Friedlander M., Backes F.J., Harter P., O’Connor D.M., de la Motte Rouge T., Lorusso D., Maenpaa J., Kim J.W., Tenney M.E. (2014). Gynecologic Cancer Intergroup (GCIG) consensus review for ovarian germ cell tumors. Int. J. Gynecol. Cancer.

[7] Elzakkers J., van der Aa M., van Altena A., de Hullu J., Harmsen M. (2019). Further insights into the role of tumour characteristics in survival of young women with epithelial ovarian cancer. Gynecol. Oncol..

[8] Pérez-Quintanilla M., del Real-Ordoñez S., Gallardo-Alvarado L., Leon D.C.-D. (2020). Fertility-sparing treatment for epithelial ovarian cancer: A literature review. Chin. Clin. Oncol..

[9] Canlorbe G., Chabbert-Buffet N., Uzan C. (2021). Fertility-Sparing Surgery for Ovarian Cancer. J. Clin. Med..

[10] Hedbäck N.E., Karlsen M.A., Høgdall C.K., Rosendahl M. (2018). Survival of selected patients with ovarian cancer treated with fertility-sparing surgery. Reprod. Biomed. Online.

[11] Kempf E., Desamericq G., Vieites B., Diaz-Padilla I., Calvo E., Estevez P., Garcia-Arreza A., Martinez-Maestre M., Duran I. (2016). Clinical and pathologic features of patients with non-epithelial ovarian cancer: Retrospective analysis of a single institution 15-year experience. Clin. Transl. Oncol..

[12] Green D.M., Sklar C.A., Boice J.D., Mulvihill J.J., Whitton J.A., Stovall M., Yasui Y. (2009). Ovarian Failure and Reproductive Outcomes after Childhood Cancer Treatment: Results from the Childhood Cancer Survivor Study. J. Clin. Oncol..

[13] van Dorp W., Haupt R., Anderson R.A., Mulder R.L., van den Heuvel-Eibrink M.M., van Dulmen-den Broeder E., Su H.I., Winther J.F., Hudson M.M., Levine J.M. (2018). Reproductive Function and Outcomes in Female Survivors of Childhood, Adolescent, and Young Adult Cancer: A Review. J. Clin. Oncol..

[14] Overbeek A., van den Berg M.H., van Leeuwen F.E., Kaspers G.J., Lambalk C.B., van Dulmen-den Broeder E. (2017). Chemotherapy-related late adverse effects on ovarian function in female survivors of childhood and young adult cancer: A systematic review. Cancer Treat. Rev..

[15] Poorvu P.D., Frazier A.L., Feraco A.M., E Manley P., Ginsburg E.S., Laufer M.R., LaCasce A.S., Diller L.R., Partridge A.H. (2019). Cancer Treatment-Related Infertility: A Critical Review of the Evidence. JNCI Cancer Spectr..

[16] Lopes F., Spears N., Anderson R. (2016). Effects of chemotherapeutic treatment on female reproductive function. Curr. Trends Clin. Embryol..

[17] Sessa C., Schneider D.T., Planchamp F., Baust K., Braicu E.I., Concin N., Godzinski J., McCluggage W.G., Orbach D., Pautier P. (2020). ESGO–SIOPE guidelines for the management of adolescents and young adults with non-epithelial ovarian cancers. Lancet Oncol..

[18] Bildik G., Akin N., Senbabaoglu F., Sahin G.N., Karahuseyinoglu S., Ince U., Taskiran C., Selek U., Yakin K., Guzel Y. (2015). GnRH agonist leuprolide acetate does not confer any protection against ovarian damage induced by chemotherapy and radiation in vitro. Hum. Reprod..

[19] Chan J.L., Wang E.T. (2017). Oncofertility for women with gynecologic malignancies. Gynecol. Oncol..

[20] Solheim O., Tropé C., Rokkones E., Kærn J., Paulsen T., Salvesen H., Hagen B., Vereide A., Fosså S. (2015). Fertility and gonadal function after adjuvant therapy in women diagnosed with a malignant ovarian germ cell tumor (MOGCT) during the “cisplatin era”. Gynecol. Oncol..

[21] Chow E.J., Stratton K.L., Leisenring W.M., Oeffinger K.C., A Sklar C., Donaldson S.S., Ginsberg J.P., Kenney L.B., Levine J.M., Robison L.L. (2016). Pregnancy after chemotherapy in male and female survivors of childhood cancer treated between 1970 and 1999: A report from the Childhood Cancer Survivor Study cohort. Lancet Oncol..

[22] Hartman M., Liu J., Czene K., Miao H., Chia K.S., Salim A., Verkooijen H.M. (2013). Birth rates among female cancer survivors: A population-based cohort study in Sweden. Cancer.

[23] Gershenson D.M. (2012). Treatment of Ovarian Cancer in Young Women. Clin. Obstet. Gynecol..

[24] Rodriguez-Wallberg K.A., Jiang Y., Lekberg T., Nilsson H.P. (2023). The Late Effects of Cancer Treatment on Female Fertility and the Current Status of Fertility Preservation—A Narrative Review. Life.

[25] Smith K.L., Gracia C., Sokalska A., Moore H. (2018). Advances in Fertility Preservation for Young Women with Cancer. Am. Soc. Clin. Oncol. Educ. Book.

[26] Di Tucci C., Galati G., Mattei G., Chinè A., Fracassi A., Muzii L. (2022). Fertility after Cancer: Risks and Successes. Cancers.

[27] Oktem O., Kim S.S., Selek U., Schatmann G., Urman B. (2018). Ovarian and Uterine Functions in Female Survivors of Childhood Cancers. Oncologist.

[28] Santos M.L., Pais A.S., Santos T.A. (2021). Fertility preservation in ovarian cancer patients. Gynecol. Endocrinol..

[29] Liu D., Cai J., Gao A., Wang Z., Cai L. (2020). Fertility sparing surgery vs radical surgery for epithelial ovarian cancer: A meta-analysis of overall survival and disease-free survival. BMC Cancer.

[30] Anderson R.A., Brewster D.H., Wood R., Nowell S., Fischbacher C., Kelsey T.W., Wallace W.H.B. (2018). The impact of cancer on subsequent chance of pregnancy: A population-based analysis. Hum. Reprod..

[31] Ratanasrithong P., Benjapibal M. (2017). Pregnancy Outcomes after Conservative Surgery for Early-Stage Ovarian Neoplasms. Asian Pac. J. Cancer Prev..

[32] Brady P.C., Forman E.J. (2021). An oncofertility prediction tool? Forecasting fertility after cancer. Fertil. Steril..

[33] Anderson R.A., Clatot F., Demeestere I., Lambertini M., Morgan A., Nelson S.M., Peccatori F., Cameron D. (2021). Cancer survivorship: Reproductive health outcomes should be included in standard toxicity assessments. Eur. J. Cancer.

[34] Cromi A., Bogani G., Uccella S., Casarin J., Serati M., Ghezzi F. (2014). Laparoscopic fertility-sparing surgery for early stage ovarian cancer: A single-centre case series and systematic literature review. J. Ovarian Res..

[35] Velez M.P., Richardson H., Baxter N.N., McClintock C., Greenblatt E., Barr R., Green M. (2021). Risk of infertility in female adolescents and young adults with cancer: A population-based cohort study. Hum. Reprod..

[36] Stensheim H., Cvancarova M., Møller B., Fosså S.D. (2011). Pregnancy after adolescent and adult cancer: A population-based matched cohort study. Int. J. Cancer.

[37] Matsuo K., Klar M., Youssefzadeh A.C., Mandelbaum R.S., Roman L.D., Ouzounian J.G., Wright J.D. (2022). Assessment of Severe Maternal Morbidity and Mortality in Pregnancies Complicated by Cancer in the US. JAMA Oncol..

[38] Haggar F.A., Pereira G., Preen D., Holman C.D., Einarsdottir K. (2014). Adverse Obstetric and Perinatal Outcomes following Treatment of Adolescent and Young Adult Cancer: A Population-Based Cohort Study. PLoS ONE.

[39] Rubens M., Ramamoorthy V., Saxena A., McGranaghan P., Appunni S., Ahmed A., Zhang Z., Burchfield S., Tonse R., Veledar E. (2022). Burden of maternal and fetal outcomes among pregnant cancer survivors during delivery hospitalizations in the United States. Sci. Rep..

[40] Wu P., Jordan K.P., Chew-Graham C.A., Mohamed M.O., Barac A., Lundberg G.P., Chappell L.C., Michos E.D., Maas A.H., Mamas M.A. (2021). In-Hospital Complications in Pregnant Women with Current or Historical Cancer Diagnoses. Mayo Clin. Proc..

